# Evolutionary Selection of the Nuclear Localization Signal in the Viral Nucleoprotein Leads to Host Adaptation of the Genus *Orthobornavirus*

**DOI:** 10.3390/v12111291

**Published:** 2020-11-11

**Authors:** Ryo Komorizono, Yukiko Sassa, Masayuki Horie, Akiko Makino, Keizo Tomonaga

**Affiliations:** 1 Laboratory of RNA Viruses, Department of Virus Research, Institute for Frontier Life and Medical Sciences, Kyoto University, Kyoto 606-8507, Japan; komorizono.ryo.6x@kyoto-u.ac.jp (R.K.); horie.masayuki.3m@kyoto-u.ac.jp (M.H.); 2 Laboratory of RNA Viruses, Department of Mammalian Regulatory Network, Graduate School of Biostudies, Kyoto University, Kyoto 606-8507, Japan; 3 Laboratory of Veterinary Infectious Disease, Tokyo University of Agriculture and Technology, Tokyo 183-8509, Japan; sassa_y@cc.tuat.ac.jp; 4 Hakubi Center for Advanced Research, Kyoto University, Kyoto 606-8507, Japan; 5 Department of Molecular Virology, Graduate School of Medicine, Kyoto University, Kyoto 606-8507, Japan

**Keywords:** bornavirus, host adaptation, viral evolution, nuclear transport

## Abstract

Adaptation of the viral life cycle to host cells is necessary for efficient viral infection and replication. This evolutionary process has contributed to the mechanism for determining the host range of viruses. Orthobornaviruses, members of the family *Bornaviridae*, are non-segmented, negative-strand RNA viruses, and several genotypes have been isolated from different vertebrate species. Previous studies revealed that some genotypes isolated from avian species can replicate in mammalian cell lines, suggesting the zoonotic potential of avian orthobornaviruses. However, the mechanism by which the host specificity of orthobornaviruses is determined has not yet been identified. In this study, we found that the infectivity of orthobornaviruses is not determined at the viral entry step, mediated by the viral glycoprotein and matrix protein. Furthermore, we demonstrated that the nuclear localization signal (NLS) sequence in the viral nucleoprotein (N) has evolved under natural selection and determines the host-specific viral polymerase activity. A chimeric mammalian orthobornavirus, which has the NLS sequence of avian orthobornavirus N, exhibited a reduced propagation efficiency in mammalian cells. Our findings indicated that nuclear transport of the viral N is a determinant of the host range of orthobornaviruses, providing insights into the evolution and host adaptation of orthobornaviruses.

## 1. Introduction

There are various barriers interfering with the viral life cycle in the cells [1]. The first barrier to viral infection is the plasma membrane. To penetrate this barrier, many viruses have evolved to use specific receptor molecules on the cell surface for fusion with the membrane and entry into the cytoplasm [2,3]. Even after entry, another host defense system, innate immunity, that rapidly detects invading pathogens and eliminates them by producing antiviral proteins, such as interferons and cytokines, confronts viruses [4]. To withstand this attack, viruses have evolved diverse strategies that can block the induction of innate immune responses and suppress the expression of antiviral proteins [5]. Adaptation of viral life cycles to host cells has repeatedly occurred to evade host defense barriers, allowing them to replicate efficiently in cells. This coevolution between viruses and hosts, therefore, leads to the adaptation of viruses to their animal hosts, contributing to the determination of viral host specificity [6].

Members of the genus *Orthobornavirus*, in the family *Bornaviridae*, are non-segmented, negative-strand RNA viruses that undergo transcription and replication in the cell nucleus. Several genotypes of orthobornaviruses have been isolated from different vertebrate species, such as mammals, birds and reptiles, and share a common genomic structure [7,8]. Their genomes encode at least six proteins; nucleoprotein (N), X protein, phosphoprotein (P), matrix protein (M), glycoprotein (G), and RNA-dependent RNA polymerase (L). N, P, and L proteins consist of the viral polymerase complex and are major components of the viral ribonucleoproteins (RNPs) [9]. M protein is located inside the viral membrane to form viral particles by coating the viral RNPs [10]. G protein is the viral envelope glycoprotein, which mediates the viral entry to host cells [11]. X protein is a negative regulator for viral polymerase and is involved in the regulation of the subcellular localization of viral RNPs by interacting with P protein [12].

A prototypical orthobornavirus is Borna disease virus 1 (BoDV-1), a mammalian orthobornavirus. BoDV-1 is the etiological agent of a neurological disease called Borna disease in horses and sheep and was also recently discovered to be associated with fatal encephalitis in humans [13,14,15,16]. On the other hand, some orthobornaviruses isolated from avian species have been established as causative agents of proventricular dilatation disease (PDD) in birds, mainly *Psittaciforme* [17,18]. Currently, more than 20 different genotypes of orthobornavirus have been isolated from various animal hosts, including humans, giving rise to a risk of the zoonotic potential of these viruses.

In a previous study, we reported that the genotypes of orthobornaviruses are subdivided into three clades [19]: clade one comprises mammalian orthobornaviruses, such as BoDV-1 and variegated squirrel bornavirus 1 (VSBV-1), a squirrel-derived zoonotic virus [20]. Clade two contains avian-derived orthobornaviruses, including parrot bornavirus 5 (PaBV-5), which appears to be associated with feather picking disease [21], and munia bornavirus 1 (MuBV-1). Clade three orthobornaviruses include two major etiological agents of PDD, PaBV-2 and PaBV-4; infections with these viruses have been reported worldwide [22,23,24]. Although infections with clade two and clade three orthobornaviruses have been found only in avian species, interestingly, mammalian cells have been shown to be permissive for infection with clade two viruses in a culture system [25,26]. These findings suggest the possibility of the zoonotic potential of clade two orthobornaviruses. However, the mechanisms by which the host specificity and adaptation of orthobornaviruses are determined have not yet been identified.

In this study, to understand the virus–host adaptation mechanism of orthobornaviruses, we infected both mammalian and avian cell lines with viruses from the three clades and sought to determine at which stage of the life cycle their host specificity is limited. As shown in previous studies [25,26], we showed that a clade two avian orthobornavirus, MuBV-1, can infect and replicate in mammalian cells. We also found by using a BoDV-1 pseudotype assay that the nonsusceptibility of mammalian cells to clade three orthobornaviruses is not determined at the stage of viral entry, mediated by the viral G and M. Furthermore, sequence comparison and dN/dS analyses revealed that the nuclear localization signal (NLS) region in the viral N but not those in other viral proteins seems to have evolved under natural selection pressure, resulting from the nuclear replication of orthobornaviruses. We also demonstrated that the NLS of N protein (N-NLS) sequence of clade three orthobornavirus affects the host cell-dependent polymerase activity and propagation efficiency of a chimeric BoDV-1 in mammalian cells. Our findings suggested that efficient nuclear transport of the viral N is critical to determine the host adaptation of orthobornaviruses, providing a better understanding of the zoonotic potential as well as the evolution of orthobornaviruses in vertebrate species.

## 2. Materials and Methods

### 2.1. Cells

Vero cell lines were maintained in Dulbecco’s modified Eagle’s medium (DMEM; Thermo Fisher Scientific, Waltham, MA, USA) containing 2% heat-inactivated fetal calf serum (FCS; MP Biomedical, Santa Ana, CA, USA). Human oligodendroglioma (OL) cell lines were maintained in DMEM containing 5% heat-inactivated FCS. The Madin–Darby canine kidney (MDCK), rat glioma (C6), COS-7, DF-1, HeLa, BHK, and NIH-3T3 cell lines were maintained in DMEM containing 10% heat-inactivated FCS. The QT6 and CHO-K1 cell lines were maintained in Dulbecco’s modified Eagle’s medium/nutrient mixture F-12 (DMEM/F12; Thermo Fisher Scientific) or DMEM/F12 Ham (Thermo Fisher Scientific) containing 10% heat-inactivated FCS, respectively. Cells were maintained at 37 °C in a humidified atmosphere containing 5% CO_2_.

### 2.2. Viruses

OL cell lines persistently infected with BoDV-1 were obtained via the reverse genetics system of strain He/80, as reported previously [27]. QT6 cell lines persistently infected with each avian orthobornavirus, were obtained by establishing persistent infection with PaBV-2 strain KOKO, PaBV-4 strain 7I6, and MuBV-1 in the cells, and these cell lines were maintained under the same conditions as the corresponding parental cell lines [28]. These cells produced infectious viruses, and > 90% of the cells were infected.

### 2.3. Viral Infection

Cell-free infection and coculture infection methods were used in this study. In the cell-free infection method [29], BoDV-infected cells were suspended in FCS-free DMEM and subjected to sonication. After centrifugation of sonicated cells at 1200× *g* for 25 min at 4 °C, the supernatant was collected and stored at −80 °C. Cells were inoculated with sonicated viral solution for 1 h. Then, inoculated cells were washed with FCS-free DMEM and maintained in DMEM containing FCS. In the coculture infection method, infected cells (2.0 × 10^6^ cells/dish) were cocultured with puromycin-resistant uninfected Vero cells (1.0 × 10^6^ cells/dish) in a 10 cm dish for 2 days, and then the cultures were incubated in medium containing 8.0 μg/mL puromycin for 14 days with a passage every 2 days to remove only the originally infected cells. In coculture experiments using cells other than Vero cells, mitomycin C (Wako, Osaka, Japan) was used to remove the originally infected cells. Briefly, mitomycin C solution was directly added to the culture medium of infected cells in a 10 cm dish at a final concentration of 15 μg/mL. After the addition of mitomycin C, the infected cells were incubated at 37 °C for 2 h. After washing three times with fresh culture medium, mitomycin C-treated infected cells were cocultured with uninfected cells (3.0 × 10^6^ cells/dish). Since mitomycin C-treated infected cells cause cell death, originally infected cells are eventually removed from the cocultures by a passage at a three-fold dilution every 2 days.

### 2.4. Plasmid Construction

G and M protein expression plasmids for each genotype (BoDV-1, PaBV-2, PaBV-4, PaBV-5, and MuBV-1) were constructed with pEF4A (Thermo Fisher Scientific). The G and M gene sequences of artificially synthesized VSBV-1 were inserted into the pCAGGS plasmid. The transcripts of the G genes were spliced to generate mature RNA-dependent RNA polymerase (L) mRNA [30,31]. To enhance protein expression levels from the plasmids, therefore, we deleted the splice acceptor sites in the cloned G constructs; the “AG” 3′-splice sites in the corresponding G gene sequences were replaced with “GG” in PaBV-2, PaBV-4 and PaBV-5 and with “AC” in MuBV-1. The sequences of the N or P genes of BoDV-1, PaBV-4, and MuBV-1 were inserted into pcDNA3 (Thermo Fisher Scientific). Sequences for expression plasmids of Myc-tagged or FLAG-tagged viral proteins were synthesized by PCR mutagenesis and inserted into pcDNA3. To construct the expression plasmids of N-NLS mutants, the NLS sequence (aa 3-30) of BoDV-1 N was substituted for the NLS sequence of PaBV-4. Similarly, the NLS sequence of PaBV-4 N that was substituted for BoDV-1 N was synthesized by PCR mutagenesis and inserted into pcDNA3.

### 2.5. Reverse Genetics of Recombinant BoDV-1

The reverse genetics system was implemented based on a previous study [27]. We used plasmids harboring full-length cDNA clones containing an extra transcription cassette encoding the GFP gene (pFct-He/80 PM-GFP) between the P and M gene regions. Briefly, 293T cells were transfected with either the pFct-He/80 PM-GFP plasmid or the pFct-He/80-rNLS PM-GFP plasmid and pCA-N, pCXN2-P, and pCAGGS-L using TransIT-293 Transfection Reagent (Mirus Bio, Madison, WI, USA). Three days after transfection, the transfected cells were passaged and cocultured with Vero cells. Cocultured cells were passaged every 4 days, and the rescue efficiency of recombinant BoDV-1 was evaluated by determining the number of GFP-positive foci per well.

### 2.6. Pseudotyped Assay of BoDV-1

293T cells infected with recombinant BoDV (rBoDV) lacking the G gene (rBoDV ΔG GFP), M gene (rBoDV ΔM GFP) and both the M and G genes (BoDV ΔMG GFP) were generated by the reverse genetics system, as reported previously [27,29]. These recombinant viruses encoded GFP as a reporter gene. Briefly, 293T cells were transfected with BoDV cDNA-expressing vector plasmids and helper plasmids expressing the BoDV-1 N, P, and L genes. The G gene expression plasmid pCAGGS-G or M gene expression plasmid pCAGGS-M was cotransfected with the helper plasmids to generate rBoDV ΔG- or rBoDV ΔM-infected cells, respectively. In the case of rBoDV ΔMG, M and G gene expression plasmids were cotransfected with the vector and helper plasmids. Cells were passaged 3 days post transfection. One day later, Vero cells stably expressing the G or the M and G were cocultured with transfected 293T cells and passaged every 3 days.

### 2.7. RT-PCR

Total RNA was extracted from cells by TRIzol (Thermo Fisher Scientific) and reverse transcribed using a Verso cDNA Synthesis Kit (Thermo Fisher Scientific) with random hexamers according to the manufacturer’s instructions. The primer pairs used were as follows: PaBV-2 fw: 5′-atgaattcaaagcatacctatgt-3′, PaBV-2 Rv: 5′-ttaagggctggaatggcgtatgt-3′; PaBV-4 Fw: 5′-atgaattcaaaacacacctacgt-3′, PaBV-4 Rv: 5′-ttaagggccggaatggcgtatgt-3′; MuBV-1 Fw: 5′-atgaattccaagcattcttacgt-3′, MuBV-1 Rev: 5′-ctaagagcttgaggtgcgaatgt-3′. RT-PCR was carried out using PrimeStar MAX polymerase (TaKaRa Bio, Shiga, Japan) according to the manufacturer’s instructions. Agarose gel electrophoresis was performed at a constant voltage of 100 V, and images were acquired with a BioDoc-It Imaging System (Ultra-Violet Products Ltd., Cambridge, UK).

### 2.8. Western Blot Analysis

Cultured cells were lysed with SDS sample buffer. Cell lysates were subjected to SDS-PAGE on e-PAGEL minigels (ATTO) after sonication using a BIORUPTOR II (Sonic Bio, Kanagawa, Japan). Then, proteins were transferred to membranes with a Trans-Blot Turbo PVDF Transfer Pack (Bio-Rad, Hercules, CA, USA), and membranes were blocked with Blocking One (Nacalai Tesque, Kyoto, Japan) and incubated with the primary antibodies. The antibodies used in this study were as follows: rabbit anti-BoDV-1 P; mouse anti-tubulin (Sigma-Aldrich, St. Louis, MO, USA), mouse anti-M2 FLAG tag (Merck, Darmstadt, Germany), and mouse anti-Myc tag 9E10 (Merck). After three washes with 0.05% Tween 20 in TBS, horseradish peroxidase-conjugated secondary antibodies were applied for 1 h at 37 °C. The bound antibodies were detected using an ECL Plus Western blotting detection reagents (GE Healthcare, Chicago, IL, USA), and an LAS-4000 Mini System chemiluminescence imaging system (GE Healthcare) was used for signal imaging and data collection.

### 2.9. Indirect Immunofluorescence Analysis

Cells seeded in 8-well chamber slides were fixed for 20 min with 4% paraformaldehyde after removal of the culture medium and permeabilized by incubation in PBS containing 0.25% Triton X-100 for 10 min. After permeabilization, cells were incubated with the rabbit anti-BoDV N, rabbit anti-BoDV P, mouse anti-FLAG tag, and mouse anti-Myc tag antibodies mentioned above for 1 h. This incubation step was followed by incubation with the appropriate Alexa Fluor-conjugated secondary antibodies (Thermo Fisher Scientific) and 4′,6-diamidino-2-phenylindole (DAPI; Merck). An ECLIPSE Ti confocal laser scanning microscope (Nikon, Tokyo, Japan) was used for cell immunofluorescence imaging and data collection.

### 2.10. Generation of Cells Stably Expressing the G Protein

The pMXs-Puro Retroviral Vector (Cell Biolabs Inc., San Diego, CA, USA) was used to generate cell lines with stable G protein expression. The G genes of each genotype were inserted in the multiple cloning site of the pMXs plasmid, and retroviral vectors were produced using the Platinum-GP Retroviral Packaging Cell Line (Cell Biolabs) and pMD2.G according to the manufacturer’s instructions for the expression of vesicular stomatitis virus G protein. Three days post transfection, the supernatants were collected as the retrovirus-containing solution and added to each cell line (OL, QT6, DF-1, and 3T3 cells). Cells permanently inoculated with retrovirus were selected in medium containing puromycin (8 µg/mL).

### 2.11. Syncytium Formation Assay

Cells stably expressing the G protein were seeded (5.0 × 10^5^ cells) in a 6-well plate, and the culture medium was removed and washed 2 times with 37 °C PBS after 24 h. Then, 5.0 mL of D-PBS+ (pH 5.0; Nacalai Tesque) containing Mg^2+^ and Ca^2+^ was added to the cells. After incubation at 37 °C for 5 min, the cells were washed 3 times with DMEM and cultured at 37 °C for 90 min in fresh medium. The culture medium was removed, and the cells were then fixed with 4% paraformaldehyde and visualized and imaged with a microscope (Nikon).

### 2.12. Minireplicon Assay

BoDV-1 and PaBV-4 minireplicon assays were performed according to the protocol described previously [32,33]. Briefly, recombinant BoDV-1 or PaBV-4 RNPs consisting of artificial minigenome antisense viral RNAs and the viral polymerase complex (N, P and L proteins) were reconstituted by transfection into cells with a Pol II-driven minigenome plasmid carrying the *Gaussia* luciferase gene and helper plasmids expressing the N, P, and L genes from BoDV-1 or PaBV-4. The reconstituted recombinant viral RNPs transcribes mRNAs encoding *Gaussia* luciferase gene by the activity of the polymerase complex. Then, 48 or 72 h post transfection, *Gaussia* luciferase activity was measured with a BioLux luciferase assay kit (New England BioLabs, Ipswich, MA, USA) and normalized to the corresponding WST-1 activity (TaKaRa) according to the manufacturer’s instructions.

### 2.13. Quantitative Real-Time RT-PCR (qRT-PCR)

Total RNA was extracted from infected cells and reverse transcribed using a Verso cDNA Synthesis Kit (Thermo Scientific) with BoDV-1 genome-specific or PaBV-4 genome-specific primers. The primers used for RT-qPCR were as follows: BoDV-1 RT primer: 5′-tgttgcgctaacaacaaaccaatcac-3′, BoDV-1 Fw: 5′-atgcattgacccaaccggta-3′, BoDV-1 Rv: 5′-atcattcgatagctgctcccttc-3′. qRT-PCR assays were carried out using SYBR Green Real-time PCR Master Mix (Toyobo, Osaka, Japan) in a Rotor-Gene Q System (Qiagen, Hilden, Germany).

### 2.14. Amino Acid Conservation Analysis

To identify mutations in viruses of the 3 clades, amino acid conservation scores were calculated by ConSurf [34]. Amino acid sequences of viral genes (N, P, M, G, and L) of BoDV-1 (accession no. AB258389), BoDV-2 (AJ311524), VSBV-1 (LN713680), PaBV-5 (LC120625), CnBV-2 (KC464478), MuBV-1, PaBV-2 (KC464478), PaBV-4 (JX065209), and PaBV-7 (JX065209) were aligned by MAFFT [35]. For analysis of the L gene, the sequence was aligned to PaBV-7 because the sequence of PaBV-7 is only partially determined. After multiple alignments, the scores were calculated with the maximum likelihood paradigm.

### 2.15. dN/dS Analysis

The amino acid sequences of the N, P, M, G and L proteins of BoDV-1 (AB258389) and PaBV-4 (JX065209) were aligned by MAFFT. The aligned sequences were divided into 12-residue segments, and nonsynonymous and synonymous substitution rates (dN/dS ratios: ω) were calculated by PAL2NAL [36]. The normalized dN/dS values for each codon were calculated using the complete coding sequences of the N genes of BoDV-1, PaBV-4 and MuBV-1 by the HyPhy package [37,38].

## 3. Results

### 3.1. Differential Infectivity of Avian Orthobornaviruses in Mammalian Cells

**Figure 1:**
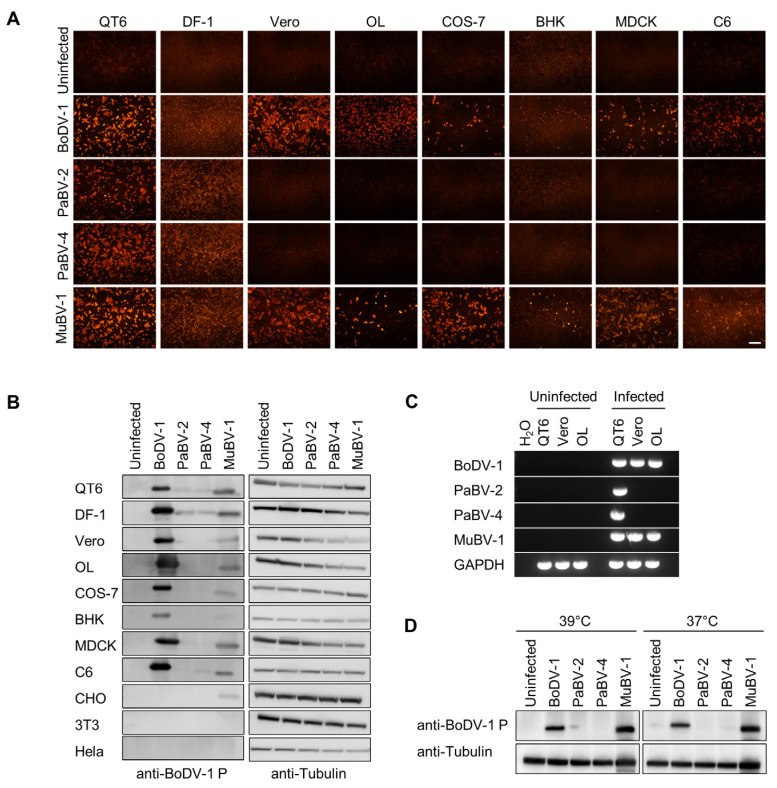
Clade-dependent host specificity of orthobornaviruses. (**A**) Host cell-dependent infection of orthobornaviruses. Orthobornaviruses from each clade (clade 1, Borna disease virus 1 (BoDV-1); clade 2, munia bornavirus 1 (MuBV-1); clade 3, parrot bornavirus 2 (PaBV-2) and PaBV-4) were inoculated into the indicated cell lines, and 14 days after infection, indirect immunofluorescence assay (IFA) was performed with an anti-BoDV-1 P antibody. Bars, 100 µm. (**B**) Detection of P antigens in orthobornavirus-infected cell lines by Western blotting. Cell lysates were collected 14 days post infection. Anti-Tubulin was used as the loading control. (**C**) Detection of viral RNAs by RT-PCR. Total RNA was extracted from inoculated cells, and RT-PCR was performed by using M-specific primers for each genotype. (**D**) Temperature-dependent replication of orthobornaviruses in mammalian cells. Vero cells were inoculated with clade 1, clade 2 and clade 3 viruses and incubated at 37 or 39 °C, and viral replication was detected by Western blotting using an anti-BoDV-1 P antibody.

Previous studies demonstrated that some clade two orthobornaviruses, including canary bornavirus 2 (CnBV-2) and estrildid finch bornavirus 1, can replicate in a mammalian cell line, albeit at very low levels, while those in clade three, which includes the main causal agents of PDD, cannot [25,26]. To understand the mechanism underlying the limitation of avian orthobornavirus replication in mammalian cells, we first evaluated the susceptibilities of several mammalian (Vero, OL, COS-7, BHK, MDCK, and C6) and avian (QT6 and DF-1) cell lines to infection with clade one (BoDV-1), clade two (MuBV-1) and clade three (PaBV-2 and PaBV-4) orthobornaviruses. Fourteen days after inoculation, the expression of viral proteins and RNAs was detected by indirect immunofluorescence assay (IFA) and Western blotting using an anti-BoDV-1 P antibody and by RT-PCR using species-specific M primers, respectively. As shown in Figure 1A,B, BoDV-1 replicated in both avian and mammalian cells. In addition, MuBV-1, a clade two orthobornavirus, but not the clade three orthobornaviruses PaBV-2 and PaBV-4, seemed to replicate efficiently in both the avian and mammalian cell lines we tested (Figure 1A,B). In Western blot analyses (Figure 1B), we additionally used CHO, NIH-3T3 and HeLa cells, which have been reported to be not susceptible to BoDV-1 infection, as controls and found that MuBV-1 shows low infectivity only for CHO cells [39,40]. RT-PCR using specific primers confirmed the replication of MuBV-1 in mammalian cell lines (Figure 1C).

Previous studies showed that avian influenza viruses are adapted for growth at the high temperature (approximately 40 °C) of the avian enteric tract and that the viral glycoproteins and polymerase proteins restrict viral replication and spread in human proximal airways, which are maintained at a cooler temperature [41,42]. Therefore, we evaluated whether avian orthobornaviruses are also adapted to replicate at the high temperature of avian cellular microenvironments. We conducted an infectivity assay of avian viruses in mammalian cells at a higher temperature. However, clade three PaBV-2 and PaBV-4 could not replicate in mammalian cells even at 39 °C (Figure 1D), suggesting that temperature does not affect the host specificity of avian orthobornaviruses.

### 3.2. The Cell Entry Step Is Not the Determinant of the Host Range Restriction of Orthobornaviruses

**Figure 2:**
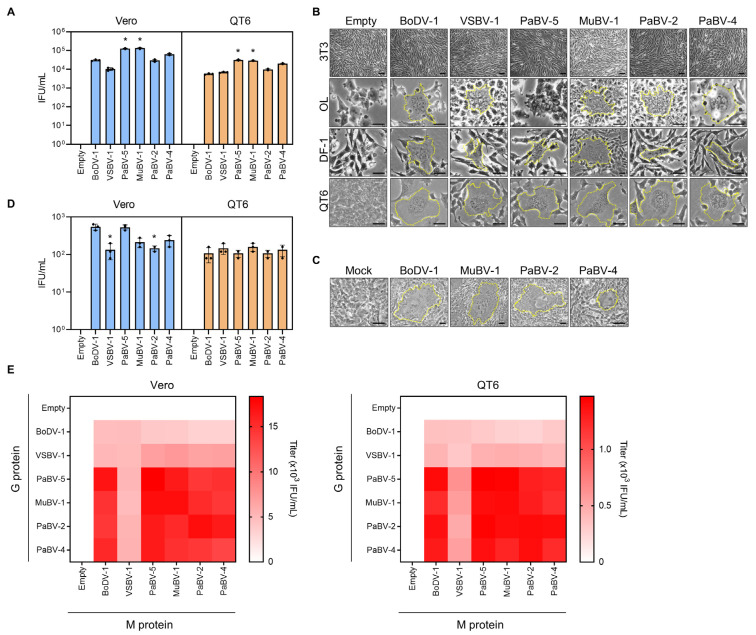
The host specificity of orthobornaviruses is not determined by the cell entry step. (**A**) Infectivity of G-pseudotyped recombinant BoDV-1 (rBoDV-1s) in Vero (blue) and QT6 cells (orange). Plasmids expressing the G protein from each genotype were transfected into 293T cells persistently infected with rBoDV ΔG GFP, and the produced G-pseudotyped BoDV-1s were inoculated into the cell lines. GFP-positive cells were counted by fluorescence microscopy at 3 days post infection. (**B**) Orthobornavirus G protein-mediated syncytium formation. Cell lines expressing glycoprotein (G) from the indicated genotypes were treated with a pH 5.0 solution, and syncytium formation was induced. The area enclosed by the yellow dotted lines indicates syncytia. Bars, 30 µm. (**C**) Syncytium formation of orthobornavirus-infected cells. QT6 cells infected with BoDV-1, PaBV-2, PaBV-4 and MuBV-1 were treated with a pH 5.0 solution, and syncytium formation was induced. Bars, 30 µm. (**D**) Infectivity of M-pseudotyped rBoDV-1s in Vero (blue) and QT6 cells (orange). The method was the same as that used in the G protein pseudotype assay. Plasmids expressing the matrix protein (M) from each genotype were transfected into 293T cells persistently infected with rBoDV ΔM GFP, and the produced viruses were inoculated into the cells. (**E**) Infectivity of MG-pseudotyped rBoDV-1s. Each combination of M and G expression plasmids from the genotypes were transfected into 293T cells persistently infected with rBoDV ΔMG GFP, and the produced viruses were inoculated into Vero (left) and QT6 (right) cells. The X and Y axes show the M and G genotypes of transfected plasmids used for generating the pseudotype viruses, respectively. Infectivity titers of each pseudotyped rBoDV-1 are indicated by color. The titers corresponding to the heat map colors are indicated. All experiments were performed with 3 biologically independent replicates. The values are expressed as the means ± SEs. Significance was analyzed by one-way ANOVA and Dunnett’s multiple comparison test. *, *p* < 0.05.

The viral life cycle contains several steps at which infection and replication in host cells can be restricted, including virion attachment and entry into target cells, intracellular transcription/replication, and de novo viral assembly and release. It has been shown that many viruses are restricted to their host range at the entry step, which is mediated by cell receptor attachment and uncoating of the viral nucleocapsid. Thus, we first investigated whether the attachment of clade three avian orthobornavirus G to the cell surface could be responsible for the restricted infection of these viruses in mammalian cells. To test this hypothesis, we employed pseudotyped BoDV-1 particles in which the G protein was replaced with those of other orthobornaviruses. Briefly, 293T cells persistently infected with G-deficient recombinant BoDV-1 (rBoDV ΔG GFP) were transfected with plasmids expressing orthobornavirus G constructs, and pseudotyped BoDV-1 particles were obtained from the supernatant of the transfected cells [27]. To generate G expression plasmids, the G gene of clade one VSBV-1 was artificially synthesized based on published sequences. As shown in Figure 2A, Vero cells were efficiently infected with all types of pseudotyped BoDV-1 particles, even those expressing G genes from clade three avian orthobornaviruses, indicating that the attachment step may not be a determinant of avian orthobornavirus infection of mammalian cells. Interestingly, G proteins of clade two avian orthobornaviruses induced similar or increased viral titers compared to those of mammalian orthobornaviruses in both mammalian and avian cells (Figure 2A).

To verify that the G proteins of orthobornaviruses can mediate membrane fusion in both mammalian and avian cells, we also performed a syncytium formation assay using cells stably expressing orthobornavirus G proteins (Figure 2B). Treatment of cells with low pH induces cell fusion to cause syncytium formation mediated by G proteins [39,40]. Mammalian (NIH-3T3 and OL) and avian (DF-1 and QT6) cell lines expressing each G protein (Figure 2B) and QT6 cells infected with BoDV-1, MuBV-1, PaBV-2, and PaBV-4 (Figure 2C) were treated with pH 5.0 buffer for 5 min, and syncytium formation was observed by light microscopy. As shown in Figure 2B, syncytium formation was induced in all cell lines stably expressing G proteins, except for NIH-3T3 cells, which are not susceptible to orthobornavirus infection. Consistent with the pseudotype assay results, G proteins from clade three avian orthobornaviruses induced syncytium formation in mammalian cells (Figure 2B). Collectively, these observations indicated that the processes of viral attachment do not limit the infection of clade three orthobornaviruses into mammalian cells.

We next assessed whether the membrane fusion of M restricts the infection of clade three orthobornaviruses in mammalian cells. To this end, we generated pseudotyped BoDV-1 particles having M from other genotypes of orthobornaviruses. Similar to the results of the G pseudotype assay, 293T cells persistently infected with rBoDV ΔM GFP were transfected with plasmids expressing orthobornavirus M constructs, and M-pseudotyped BoDV-1 particles were obtained. The infection assay revealed that regardless of the origin of M, pseudotyped BoDV-1 can replicate in both mammalian and avian cells (Figure 2D). In addition, we demonstrated that pseudotyped BoDV-1s expressing G and M proteins from different genotypes in various combinations could infect both cell types (Figure 2E). Collectively, these observations suggested that the cell entry step is not the determinant of restricted clade three virus replication in mammalian cells.

### 3.3. The N-Terminal Region of Orthobornavirus N Has Undergone Positive Selection

To understand the difference in the host cell infectivity of orthobornaviruses, we next conducted comparative genomics analyses using genome sequences of orthobornaviruses and tried to detect the coding regions that underwent clade-dependent selection during evolution. The regions with evolutionary selection are expected to be the sites where mutations have accumulated to adapt to different host environments during viral evolution and are assumed to be important in determining the host specificity of viruses.

**Figure 3:**
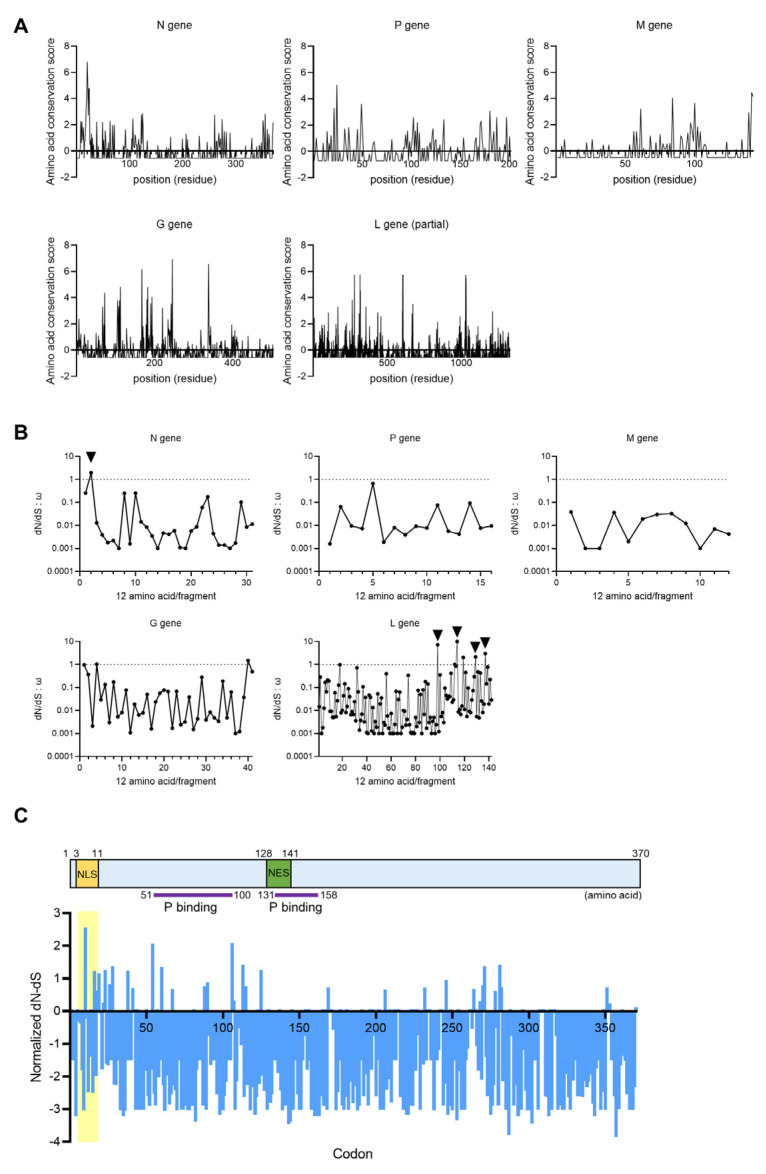
The nuclear localization signal of orthobornavirus N has undergone positive selection. (**A**) The amino acid conservation scores for each position in each viral protein were calculated by ConSurf (https://consurf.tau.ac.il/). The amino acid sequence of BoDV-1 was used as the query sequence. The lowest score indicates the most conserved site in a protein. (**B**) The nonsynonymous to synonymous substitution (dN/dS) ratios of orthobornavirus proteins. The aligned amino acid sequences of the nucleoprotein (N), phosphoprotein (P), matrix protein (M), glycoprotein (G), and RNA-dependent RNA polymerase (L) proteins of BoDV-1 and PaBV-4 were divided into 12-residue segments, and the dN/dS ratios (ω) were calculated. (**C**) The normalized dN-dS values of each codon were calculated using the complete coding sequences of the N genes of BoDV-1, PaBV-4 and MuBV-1.

To detect the evolutionarily pressured regions, we first analyzed the similarity of amino acid sequences of viral proteins among the three clades of orthobornaviruses. After multiple alignment, the similarity of each residue was calculated based on the sequence of BoDV-1 as the query sequence by the ConSurf tool with the maximum likelihood paradigm [34]. The amino acid conservation scores calculated by ConSurf are the indexes of evolutionary conservation at each query site. The low score indicates a conserved position in all viral proteins except for X. As shown Figure 3A, higher scores were found within the N, G and L genes than in the other genes, suggesting that these genes may have accumulated mutations during evolution.

We next determined the nonsynonymous to synonymous substitution (dN/dS) ratio of coding sequences of clade one and three orthobornavirus genes to measure selective pressure on amino acids (aa). A dN/dS ratio of 1.0 indicates neutral drift, whereas dN/dS ratios of <1.0 and >1.0 suggest purifying and diversifying selection, respectively. We estimated the dN/dS ratio for each 12-aa stretch of the viral proteins of BoDV-1 and PaBV-4 by using the PAML package [36]. As shown in Figure 3B, regions under diversifying selection (dN/dS > 1.0), which seem to have acquired more dominant mutations than other regions, were found at the N- and C-terminal regions of N and L, respectively, suggesting that these regions may have a positive effect on viral evolution. While we could not find any functional motifs in regions where the dN/dS ratio was greater than 1.0 in L, the N-terminal sequence of N contains a previously identified NLS (P_3_KRRLVDDA_11_) [43]. Therefore, we further focused on the sequence of N and calculated the distribution of the normalized dN-dS values for each codon using the N proteins of BoDV-1 (clade one), MuBV-1 (clade two) and PaBV-4 (clade three). As shown in Figure 3C, the positive values indicating positive selection seemed to be particularly accumulated in the N-terminal region containing the NLS, suggesting that this region may be involved in the clade dependent selection that occurred during orthobornavirus evolution.

### 3.4. PaBV-4 N Reveals Weak Nuclear Localization Activity in Mammalian Cells

**Figure 4:**
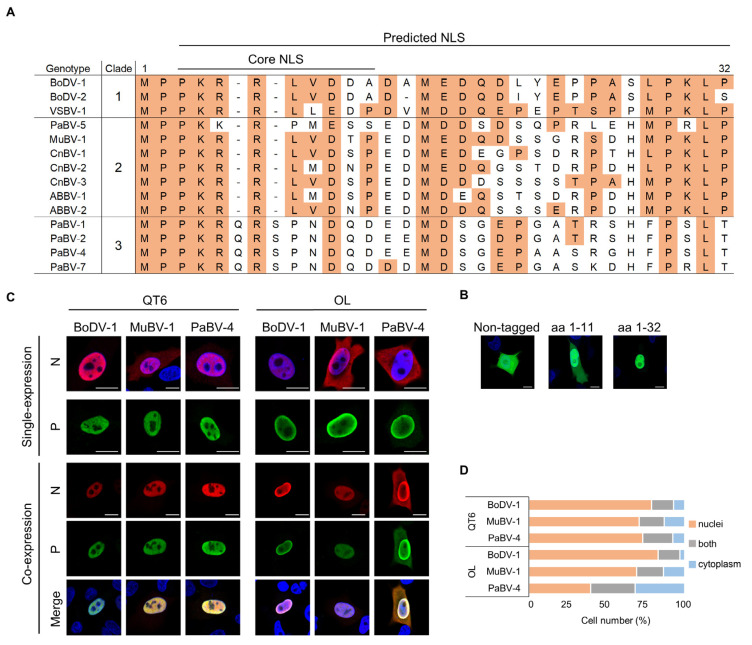
Comparison of the nuclear localization activity of orthobornavirus N proteins. (**A**) Alignment of NLS sequences of N proteins among orthobornavirus genotypes. The nuclear localization signal (NLS) regions (aa 1-32) predicted by cNLS Mapper were aligned. The residues identical to those of BoDV-1 are shown in yellow boxes. (**B**) Nuclear localization activity of BoDV-1 N-NLS. Human oligodendroglioma (OL) cells were transfected with plasmids encoding BoDV-1 N-NLS (aa 1-11 or 1-32)-fused tandem GFP, and GFP fluorescence was observed at 48 h post transfection. Bars, 10 μm. (**C**) Subcellular localization of the N and P proteins of BoDV-1, MuBV-1 and PaBV-4 in avian and mammalian cells. Myc-tagged N and FLAG-tagged P expression plasmids were transfected into QT6 and OL cells alone or together. The subcellular localization of N and P was detected by immunostaining 48 h after transfection. Representative images of transfected cells are shown. Bars, 10 μm. (**D**) Subcellular localization of N in avian and mammalian cells co-transfected with N and P expression plasmids. QT6 and OL cells were transfected with plasmids expressing the N protein and FLAG-tagged P protein from BoDV-1 (clade 1), MuBV-4 (clade 2) and PaBV-4 (clade 3). The subcellular localization of N proteins was analyzed by fluorescence immunostaining at 48 h post transfection by confocal microscopy. N protein localization (nuclear, cytoplasmic, or both) was assessed (200 cells, *n* = 3), and the percentage was determined.

To understand the clade-specific host range, we observed the N-termini of orthobornavirus N protein in detail. Alignment of the regions of orthobornavirus N proteins revealed that clade one and two viruses but not clade three viruses exhibited conserved NLS sequences (Figure 4A). Interestingly, insertions of two aa (Q6/S8) and some unique substitutions were found in the core NLS of clade three viruses (Figure 4A). cNLS Mapper [44,45], which can predict importin α-dependent NLSs, showed that the NLS of BoDV-1 N may have a much longer sequence (aa 3-32) than expected. In fact, a tandem GFP molecule fused with a 32-aa stretch of the N-terminus of BoDV-1 N was completely localized in the nucleus of transfected cells, while weak cytoplasmic retention of GFP was found in cells transfected with GFP containing only the core NLS sequence (aa 1-11) (Figure 4B), suggesting that the N-terminal 32 aa, in which the values indicating positive selection predicted by dN/dS analysis were accumulated, is an expanded NLS in orthobornavirus N proteins.

We next compared the nuclear localization ability of orthobornavirus N proteins in avian and mammalian cells in the presence or absence of P, because previous studies demonstrated that interaction with P facilitates nuclear retention of N by masking the nuclear export signal on N [46,47]. We generated myc- and FLAG-tagged N and P constructs, respectively, from BoDV-1, MuBV-1, and PaBV-4 and transfected them into OL and QT6 cells. As shown in Figure 4C, whereas BoDV-1 N showed clear nuclear localization in both mammalian and avian cells by single expression, the N proteins of MuBV-1 and PaBV-4 were localized to both the cytoplasm and nucleus. Interestingly, co-expression with P induced nuclear accumulation of MuBV-1 N in mammalian cells (Figure 4C,D). However, PaBV-4 N retained its cytoplasmic localization even in the presence of P in OL cells (Figure 4C,D), suggesting the weak nuclear localization activity of PaBV-4 in mammalian cells.

### 3.5. The NLS in PaBV-4 N Reduces rBoDV-1 Replication in Mammalian Cells

**Figure 5:**
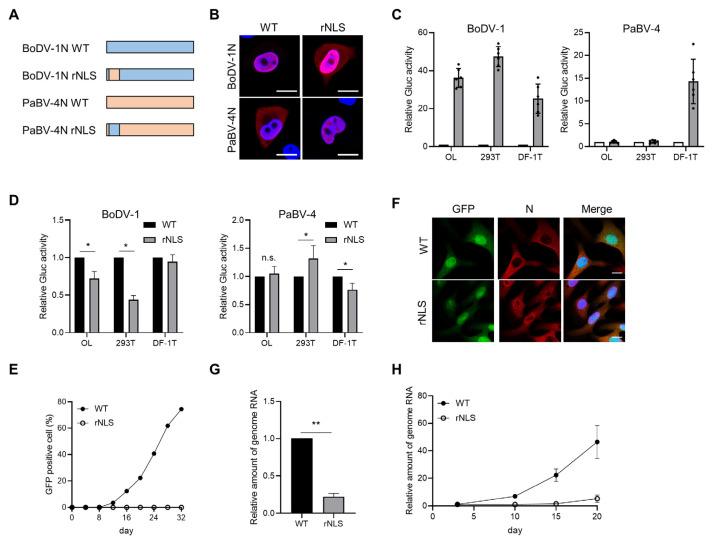
BoDV-1 N NLS enhances the polymerase activity of PaBV-4 in mammalian cells. (**A**) Schematic diagram of constructed chimeric N proteins. (**B**) Subcellular localization of chimeric N proteins in OL cells. The subcellular localization of each protein was assessed by confocal microscopy. Bars, 10 μm. (**C**) BoDV-1 and PaBV-4 minireplicon assays. The minireplicon plasmid encoding *Gaussia* luciferase (Gluc) was co-transfected with helper plasmids into the indicated cell lines. Gluc activity was measured 72 h post transfection. The relative ratio of Gluc activity to that in negative control cells not transfected with the L expression plasmid is shown. (**D**) Effects of N-NLS substitution on the minireplicon activity of BoDV-1 and PaBV-4. The relative ratio of Gluc activity using chimeric N (rNLS) construct to that of the wild-type (WT) N construct is shown. The data are presented as the means and +SEs of results from six independent experiments. One-way analysis of variance (ANOVA) and Tukey’s post hoc test were used for statistical analysis. *, *p* < 0.05. (**E**) Rescue curves of WT rBoDV-1 and chimeric rBoDV-1 (rNLS) by the reverse genetics system. The percentage of GFP-positive cells in Vero cells after coculture with transfected 293T cells was monitored with Tali^TM^ Image-Based Cytometer. (**F**) Establishment of cell lines persistently infected with rBoDV-1 rNLS. Vero cells infected with rBoDV-1 rNLS shown in panel (**E**) were cloned to establish the persistently infected cell lines, and IFA was performed with the anti-BoDV-1 N antibody. Bars, 10 μm. (**G**) Relative amounts of genomic RNA of rBoDV-1 rNLS in persistently infected Vero cells. Experiments were performed with six biologically independent replicates. The values are expressed as the means + SEs of the results from three independent experiments. Significance was analyzed by Student’s *t* test. **, *p* < 0.001. (**H**) Growth kinetics of WT and BoDV-1 rNLS in OL cells. Cells were infected with rBoDV-1s at a multiplicity of infection (MOI) of 0.05. The levels of genomic RNA at the indicated time points were measured by qRT-PCR. The experiment was performed with three biologically independent replicates.

The nuclear localization of N is critical for efficient intranuclear replication of orthobornaviruses. Therefore, it is conceivable that the NLS has evolved during adaptation to host species and may determine the host range. To test this hypothesis, we generated chimeric N proteins containing the NLS from viruses in different clades (Figure 5A). Interestingly, BoDV-1 N with the PaBV-4 NLS (BoDV-1N rNLS) exhibited cytoplasmic leakage of the N protein in transfected mammalian cells (Figure 5B). In contrast, PaBV-4N rNLS, which had a BoDV-1 NLS in PaBV-4 N, showed clear nuclear localization, indicating that the clade three N-NLS is an insufficient signal for nuclear targeting in mammalian cells.

Next, to investigate the effect of the nuclear targeting activity of the N-NLS on viral polymerase activity, we used BoDV-1 and PaBV-4 minireplicon systems, which reconstruct viral minigenomes expressing luciferase as a reporter gene. As shown in Figure 5C, the BoDV-1 minireplicon worked comparably in both mammalian and avian cells, while luciferase activity was not induced in mammalian cells by transfection of a PaBV-4 minireplicon set. We thus used BoDV-1N rNLS and PaBV-4N rNLS for the minireplicon systems. When BoDV-1N rNLS was employed instead of BoDV-1N WT in the BoDV-1 minireplicon assay, luciferase activity was significantly reduced in mammalian cells but not in DF-1T cells (Figure 5D). On the other hand, PaBV-4N rNLS slightly but significantly increased luciferase activity in 293T cells and reduced luciferase activity in avian cells with the PaBV-4 minireplicon. (Figure 5D). These results suggested that the host-adapted N-NLS is important for the polymerase activity of the minireplicon systems.

To understand the role of the N-NLS in orthobornavirus replication in more detail, we used a reverse genetics approach to generate a chimeric rBoDV-1 genome with the PaBV-4 N-NLS sequence, named rBoDV-1 rNLS, based on a GFP-expressing BoDV-1 vector. We transfected plasmids expressing the chimeric rBoDV-1 genome and helper viral proteins into 293T cells, and three days after transfection, cocultured the cells with Vero cells. Although wild-type (WT) rBoDV-1 exhibited an infection rate of 80% at least 30 days after coculture with Vero cells, the chimeric rBoDV-1 rNLS exhibited an infection rate of less than 1% during this period (Figure 5E), indicating that the chimeric virus showed a lower rescue efficiency than WT rBoDV-1. To investigate the viral properties, we cloned rBoDV-1 rNLS-positive Vero cells and established persistently infected cell lines (Figure 5F). We found that in cells infected with rBoDV-1 rNLS, the N protein seemed to be diffusely distributed within the nucleus (Figure 5F). In addition, rNLS virus-infected Vero cells contained significantly less genomic RNA than WT rBoDV-1-infected cells (Figure 5G). Furthermore, we inoculated WT and chimeric rNLS viral particles prepared from the persistently infected cells into OL cells at an MOI of 0.05. As shown in Figure 5H, rBoDV-1 rNLS exhibited extremely slow propagation in OL cells. Less than 5% of the cells were infected 20 days post inoculation with rBoDV-1 rNLS. These data indicate that rBoDV-1 with the PaBV-4 N-NLS exhibits reduced replication efficiency in mammalian cells.

### 3.6. Avian Importin α Increases the Polymerase Activity of the PaBV-4 Minireplicon in Mammalian Cells

**Figure 6:**
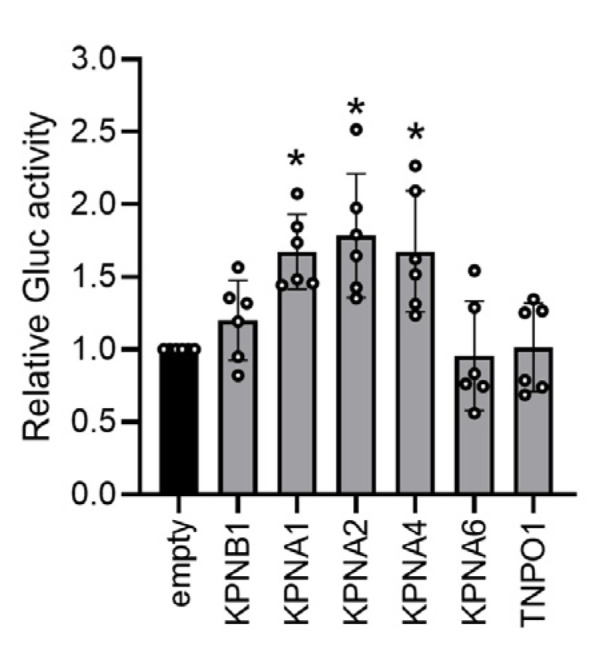
Avian importin α proteins increase the polymerase activity of the PaBV-4 minireplicon in mammalian cells. 293T cells were co-transfected with expression plasmids encoding the PaBV-4 N, P, and L proteins and the indicated chicken importin proteins. Gluc activity was measured 72 h post transfection and was normalized to WST-1 activity. The relative ratios of Gluc activity in the cells transfected with importin plasmids to those with pcDNA3 empty plasmid are shown. Each dot represents the relative ratio of an independent experiment. The data are presented as the means and +SEs of results from six independent experiments. One-way ANOVA followed by a Dunnett’s test was used for statistical analysis. *, *p* < 0.05.

Finally, we investigated whether avian host factors involved in nuclear import, such as importin α and β, can rescue the polymerase activity of the PaBV-4 minigenome in mammalian cells. We cloned avian importin proteins from QT6 cells and co-transfected them into 293T cells with a PaBV-4 minireplicon set. As shown in Figure 6, transfection of importin α family proteins (KPNA1, KPNA2 and KPNA4), but not importin β proteins, markedly upregulated luciferase activity in mammalian cells. This observation confirmed that a host species-specific N-NLS is critical for nuclear targeting of the orthobornavirus N protein and determines polymerase activity in the nucleus.

## 4. Discussion

In this study, we examined the infectivity of several genotypes of the genus *Orthobornavirus* in both mammalian and avian cell lines and showed that, as previously reported [48], viruses derived from clade three avian orthobornaviruses, such as PaBV-2 and PaBV-4, have no replication capacity in mammalian cells, although the clade two virus MuBV-1 does (Figure 1). We also demonstrated by using a recombinant BoDV-1 pseudotype assay that the infectivity of clade three orthobornaviruses is not determined at the stage of viral entry mediated by the G and M proteins (Figure 2). Furthermore, comparative sequence and dN/dS analyses of orthobornaviral proteins revealed that the NLS region of the N protein has undergone evolutionary selection pressure (Figure 3) and that the NLS sequences are involved in the host cell-dependent polymerase activity of these viruses (Figure 5). These findings suggested that efficient nuclear transport of the viral N may determine the host cell specificity or host adaptation of orthobornaviruses.

The host range and tissue tropism of viruses are determined at various steps in their intracellular life cycles. The viral entry step, including attachment to the cell surface and penetration into the cell, mediated by specific receptor molecules, is an initial barrier to the infection of host cells. For example, influenza A viruses recognize sialic acids in glycans on the cell surface as attachment receptors via viral hemagglutinin (HA) [49,50,51]. The binding specificities of HAs to sialic acids depend on the host species from which the virus was isolated, and avian influenza viruses preferentially bind to glycans with the sialic acid α2,3-galactose, to which the HA of human influenza viruses shows a weak binding affinity [52,53,54]. The host specificity of morbilliviruses, such as measles virus and canine distemper virus, are also determined via recognition of the host-specific signaling lymphocyte activation molecule (SLAM) as a binding receptor [55,56,57]. Receptor-dependent viral penetration is also known to contribute to susceptibility to infection with many viruses [2,58]. The binding affinity of Ebola virus glycoprotein to an endosomal protein, Niemann-Pick C1, affects the fusion ability of the virus to host membranes, which determines the host range of the virus in cultured cells [59]. These observations reveal that receptor-mediated, host- or cell-specific entry mechanisms, which constitute a major barrier to the cross-species infection of viruses, have been developed in coevolution between viruses and hosts.

Currently, the mechanism by which the host specificity of orthobornaviruses is determined is not yet understood. In this study, we found that the cell tropism of orthobornaviruses is not restricted at the stage of cell entry. Pseudotype viruses expressing either—or both—of the clade three avian orthobornavirus G and M proteins can efficiently infect mammalian cells. In addition, the syncytium formation assay showed that the G proteins of clade three avian viruses induce membrane fusion activity in not only avian but also mammalian cells. These results suggested that orthobornaviruses bind one or more conserved receptor molecule(s) that are expressed in both avian and mammalian cells to enter the cells, although the binding receptor has not yet been identified [60]. Identification of the cellular receptor(s) involved in the entry of orthobornaviruses will provide a better understanding of the host specificity and neuropathogenicity of these viruses, as well as their potential as zoonotic pathogens.

Animal viruses replicating in the cell nucleus must pass through not only the plasma membrane but also the nuclear envelope to complete their life cycles. In most cases, nuclear pore complexes (NPCs) are used to transport viral components into the nucleus through the nuclear envelope [61]. NPCs are large multiprotein complexes that function as gates for the transport of molecules into and out of the nucleus. The selectivity of molecules passing through NPCs is determined by interactions between NLSs and members of a family of soluble transport receptors, the karyopherins—for example, importins. Karyopherins interact with cargos containing NLSs to transport them to NPCs for passage through the nuclear envelope [62,63]. This system is widely used in many viruses to transport their proteins into the nucleus [64]. For example, herpes simplex virus 1 uses importin α1 for nuclear targeting of some viral proteins and the production of infectious virions [65]. It has also been reported that nuclear transport of the polymerase subunits of avian and mammalian influenza A viruses is dependent on species-specific importin α subunits [66]. In addition, nuclear transport of preintegration complexes of human immunodeficiency virus (HIV-1) is restricted in mouse cells due to the insufficient interaction of HIV-1 integrase with the murine transport system [67]. Interestingly, bioinformatic analyses demonstrated that the NLS sequences of herpesvirus terminases, which are the molecular motors for encapsidation of viral DNA into empty capsids, are evolving more rapidly than those of other viral proteins and have diversified among subfamilies [68]. Since the binding affinity between NLS and transporter receptors is important for efficient nuclear transport of viral proteins, the evolution of viral NLS sequences to maintain their affinity for host transporter receptors may be necessary for host adaptation during viral diversification.

In this study, we showed that the NLS of the orthobornavirus N protein may have evolved as a sequence involved in host adaptation. Positive selection was identified in the NLS region of the N by dN/dS analysis. Interestingly, although orthobornaviruses also have NLSs in other viral proteins, including P, X, M, and L, natural selection was only found in the N-NLS. This may show that N is a main driving factor in the transport of viral RNPs into the nucleus as a protein consisting of the nucleocapsid [69]. The sequence comparison of the regions of N-NLS among the orthobornaviruses revealed that the sequences of the clade two avian viruses, which can infect mammalian cells, are more similar to those of the clade one mammalian viruses, than to the clade three viruses. On the other hand, the nuclear localization activity of clade two MuBV-1 N seemed to be weak, since single expression of the protein localized at both the cytoplasm and nucleus in mammalian cells, similar to clade three PaBV-4-N. MuBV-1 N, but not PaBV-4 N, was however, shown to be completely transported to the nucleus when it was co-expressed with its own P in mammalian cells. Previous studies revealed that BoDV-1 P acts as a nuclear retention factor of N by masking the nuclear export signal (NES) of N via direct interaction [46,47]. This indicated that the masking of N-NES by P allows the activity of N-NLS to be observed without considering the efficiency of the nuclear transport activity by the NES, suggesting that the NLS of MuBV-1 N efficiently functions in mammalian cells, which may play a role in the infectivity of the clade two avian orthobornavirus to mammalian cells.

We showed here that the NLS sequence in the N may affect the host-dependent polymerase activities in both avian and mammalian cells by using the minireplicon system of orthobornaviruses. Furthermore, rBoDV-1 with the N-NLS from PaBV-4 significantly reduced the replication efficiency in mammalian cells. In addition, the polymerase activity of the PaBV-4 minireplicon was increased by transduction of importin α subunits from birds in mammalian cells. Collectively, these results indicated that the nuclear transport ability of the N mediated by the NLS determines, if not exclusively, the host specificity of the orthobornaviruses. Future development of reverse genetics for avian orthobornaviruses, particularly clade three viruses, would provide a detailed interaction between nuclear transport and host specificity.

In this study, we showed that the orthobornavirus N-NLS has evolved under natural selection and that the nuclear localization efficiency of the N is a key factor affecting the host range of the viruses. Future elucidation of the nuclear transport mechanisms of orthobornavirus RNPs, as well as identification of host factors involved in nuclear transport, will not only promote the development of antiviral drugs against orthobornaviruses but also expand our understanding of orthobornaviruses as zoonotic pathogens.
